# Artificial Intelligence’s Role in Improving Adverse Pregnancy Outcomes: A Scoping Review and Consideration of Ethical Issues

**DOI:** 10.3390/jcm14113860

**Published:** 2025-05-30

**Authors:** Mariana Nogueira, Sandra Lopes Aparício, Ivone Duarte, Margarida Silvestre

**Affiliations:** 1 Faculty of Medicine, University of Porto, 4200-319 Porto, Portugal; 2 RISE-Health, Health Research and Innovation, Faculty of Medicine, University of Porto, 4200-319 Porto, Portugal; smaparicio@med.up.pt (S.L.A.); iduarte@med.up.pt (I.D.); 3 Department of Community Medicine, Information and Health Decision Sciences (MEDCIDS), Faculty of Medicine, University of Porto, 4200-319 Porto, Portugal; 4 Faculty of Medicine, University of Coimbra, 3004-531 Coimbra, Portugal; msilvestre@fmed.uc.pt

**Keywords:** artificial intelligence, perinatal care, pregnancy outcome, ethics

## Abstract

**Background/Objectives**: Adverse pregnancy outcomes (APOs), which include hypertensive disorders of pregnancy (gestational hypertension, preeclampsia, and related disorders), gestational diabetes, preterm birth, fetal growth restriction, low birth weight, small-for-gestational-age newborn, placental abruption, and stillbirth, are health risks for pregnant women that can have fatal outcomes. This study’s aim is to investigate the usefulness of artificial intelligence (AI) in improving these outcomes and includes changes in the utilization of ultrasound, continuous monitoring, and an earlier prediction of complications, as well as being able to individualize processes and support clinical decision-making. This study evaluates the use of AI in improving at least one APO. **Methods**: PubMed, Web of Science, and Scopus databases were searched and limited to the English language, humans, and between 2020 and 2024. This scoping review included peer-reviewed articles across any study design. However, systematic reviews, meta-analyses, unpublished studies, and grey literature sources (e.g., reports and conference abstracts) were excluded. Studies were eligible for inclusion if they described the use of AI in improving APOs and the associated ethical issues. **Results**: Five studies met the inclusion criteria and were included in this scoping review. Although this review initially aimed to evaluate AI’s role across a wide range of APOs, including placental abruption and stillbirth, the five selected studies focused primarily on preterm birth, hypertensive disorders of pregnancy, and gestational diabetes. None of the included studies addressed placental abruption or stillbirth directly. The studies primarily utilized machine-learning models, including extreme gradient boosting (XGBoost) and random forest (RF), showing promising results in enhancing prenatal care and supporting clinical decision-making. Ethical considerations, including algorithmic bias, transparency, and the need for regulatory oversight, were highlighted as critical challenges. **Conclusions**: The application of these tools can improve prenatal care by predicting obstetric complications, but ethics and transparency are pivotal. Empathy and humanization in healthcare must remain fundamental, and flexible training mechanisms are needed to keep up with rapid innovation. AI offers an opportunity to support, not replace, the doctor–patient relationship and must be subject to strict legislation if it is to be used safely and fairly.

## 1. Introduction

Pregnancy is a complex period of a woman’s life. The body’s necessary adaptations to pregnancy lead to changes in maternal physiology, which can predispose the woman to developing diseases and problems. Due to various factors, both demographic and lifestyle-related, the incidence of these problems is increasing, requiring a multidisciplinary approach [1,2,3]. We are dealing with a field in which results can be unforeseen, so in developing obstetric guidelines, ways of reducing error are important. It is known that adverse outcomes often occur due to system deficiencies or safety measures that are ineffective in preventing or reducing errors. However, the intention is not to exclude medical responsibility for the acts carried out [4].

Among the adverse pregnancy outcomes (APOs) that represent causes of maternal and neonatal morbidity and mortality [3], the most important are hypertensive disorders of pregnancy (HDP) (gestational hypertension, preeclampsia, and related disorders), gestational diabetes mellitus (GDM), preterm birth (PTB), fetal growth restriction (FGR), low birth weight (LBW), small-for-gestational-age newborn (SGA), placental abruption, and stillbirth [5].

Hypertensive disorders are conditions in which maternal systolic blood pressure is greater than or equal to 140 mm Hg and/or diastolic blood pressure measures greater than or equal to 90 mm Hg on two or more occasions at least four hours apart [6,7,8]. Gestational diabetes is a type of diabetes that develops during pregnancy and is diagnosed by fasting blood glucose in the first trimester ≥ 92 mg/dl, or in the 24th to 28th week of pregnancy, by performing an oral glucose tolerance test (OGTT) with 75 gr and values ≥ 92, ≥180, and ≥153 mg/dL fasting, after 1 and 2 h [9,10,11,12,13,14]. Preterm birth is defined as a baby born alive before 37 weeks. They are categorized based on gestational age: extremely preterm (less than 28 weeks), very preterm (28 to less than 32 weeks), and moderate- to late-preterm (32 to 37 weeks) [15,16,17]. Fetal growth restriction is an estimated fetal weight less than the tenth percentile for gestational age by prenatal ultrasound evaluation [18,19]. Linked to the latter concept, low birth weight is defined as the first weight recorded within hours of the birth of <2500 g. Very low birth weight (VLBW) is accepted as <1500 g, and extremely low birth weight (ELBW) is <1000 g [20]. Small-for-gestational-age describes a baby who is smaller than the usual weight for the number of weeks of pregnancy, with birth weights below the 10th percentile for babies of the same gestational age [21,22]. Placental abruption is when the placenta detaches from the uterus during pregnancy; it is a cause of bleeding and abdominal cramping and is a serious complication of pregnancy [23,24]. Stillbirth is defined as the worst of events, with the death of a fetus prior to birth at 24 weeks of gestation or more [25,26].

The exponential growth of artificial intelligence (AI) is defined as the use of neural networks, machine-learning (ML), or deep-learning methods; there has been a growing approach to its use in obstetrics. This is especially important if we want to reverse unfavorable outcomes and combat social inequalities in access to more and better healthcare [27]. AI is expected to be integrated into obstetrics in a revolutionary way, but with ethical considerations [28]. Recognizing APOs as a possibly harmful complication because they increase the risk of future cardiovascular disease [3,29], it is essential to improve long-term health outcomes [3]. Maternal–fetal mortality and morbidity can be reduced through the use of AI applications by carrying out adequate monitoring and enabling rapid detection of risk factors, as well as allowing doctors to make more assertive and timely decisions [27,30].

With research into and the application of AI to sustainable development goals (SDGs), it is expected that techniques and legal gaps will be improved, and it will be recognized that no single theory is always valid in all situations for everyone. The aim is to continue to reduce maternal mortality, which can be achieved through technological innovation and scientific and medical knowledge [31].

Despite its increasing use, particularly in the last two decades, publications related to AI in obstetrics remain outside the scope of journals in the field. Machine-learning algorithms have made it possible to make more targeted and assertive predictions and recognize patterns, which can enable more timely interventions [32].

Some ethical issues are pivotal [33]. One of them, and one of the most discussed, is bias in AI algorithms that can produce disparities in maternal care. Concerns about data privacy, security, autonomy, and informed consent imply a strategy of transparency and clear communication, but they can also contribute to accuracy and cost-effective solutions [33,34,35]. Applying AI methods can help medical practitioners in pregnancy risk assessment, which could be applied to APO prediction. Nevertheless, further studies could identify new methods with an even better prediction potential [34,36,37].

This scoping review aim is to find out how AI can improve the main adverse pregnancy outcomes, including hypertensive disorders of pregnancy (gestational hypertension, pre-eclampsia, and related disorders), gestational diabetes, preterm birth, fetal growth restriction, low birth weight, small-for-gestational-age newborns, placental abruption, and stillbirth, and how its use can prevent fatal outcomes and poor prognoses. Ethical issues are also considered, such as clinical decision-making with the use of AI, as well as autonomy and informed consent.

## 2. Materials and Methods

This scoping review was conducted in accordance with the recommendations of the Preferred Reporting Items for Systematic Reviews and Meta-Analyses extension for Scoping Reviews (PRISMA-ScR) to ensure methodological transparency and consistency [38]. The PRISMA-ScR Checklist is presented in the Supplementary Materials.

To guide the identification and selection of relevant studies, the PCC framework was applied as follows: P (Population): pregnant women; C (Concept): use of artificial intelligence; C (Context): maternal health care aiming to improve pregnancy outcomes [38].

The protocol for this scoping review was registered on the International Platform of Registered Systematic Review and Meta-analysis Protocols (INPLASY), unique ID: INPLASY202520065 (https://inplasy.com/inplasy-2025-2-0065/), accessed on 12 February 2025.

A thorough literature search was performed in the SCOPUS (Table 1), PubMed (Table 2), and Web of Science (Table 3) databases. The keywords were selected based on MeSH terms and relevant terminology used in prior reviews. Table 1, Table 2 and Table 3 list the queries as applied to each database, using combinations of AI-related and obstetric outcome terms. Google Scholar was excluded due to its non-curated nature and lack of reproducibility, which may introduce bias and reduce methodological transparency.

This scoping review included peer-reviewed original studies of any design, published in English. This review focused on studies published between 2020 and 2024 to ensure the inclusion of the most recent evidence. In this period, the application of artificial intelligence in healthcare, particularly in obstetrics, has undergone significant development, with improved technologies and growing clinical integration. To ensure methodological rigor and consistency in reporting, we excluded secondary publications, such as systematic reviews, meta-analyses, narrative reviews, commentaries, editorials, letters, case reports, books, and grey literature, including unpublished studies and conference materials [39,40,41]. Studies lacking clear reporting of relevant variables or including redundant information were excluded.

All studies identified through the computerized search of the databases were imported into the Rayyan platform (https://rayyan.ai/), accessed on 14 January 2025, a widely recognized tool in the scientific community for systematic, scoping, and narrative reviews, which was used for the selection process. Following the established inclusion criteria, two independent researchers screened the studies to ensure that only articles meeting these criteria were selected. A study’s inclusion required agreement between the two reviewers. Any discrepancies were resolved through discussion or by consulting a third reviewer until an agreement was reached.

The selection process involved eliminating duplicate articles and was conducted in three stages: the first stage examined the article titles, the second stage reviewed the abstracts, and the final stage consisted of reading the articles in their entirety.

The final scoping review reported search results and the study inclusion process in full and presented a Preferred Reporting Items for Systematic Reviews and Meta-analyses extension for scoping reviews (PRISMA-ScR) flow diagram [38].

**Figure 1:**
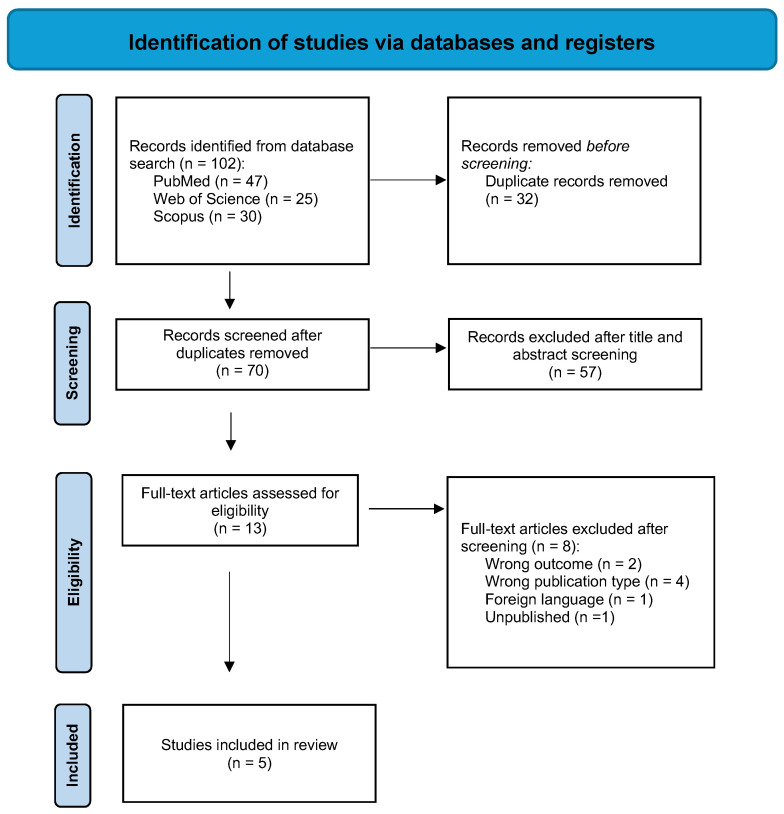
PRISMA flow diagram showing the literature method search: n, the number of articles.

A total of 102 citations were retrieved from the databases. After removing 32 duplicates, 70 citations underwent title and abstract screening. Of these, 13 proceeded to full-text screening and were assessed for eligibility. All told, eight studies were excluded for the following reasons: two for the wrong outcome, four for the wrong publication type, one for a foreign language, and one was not published yet. In total, five papers were included in the final review and underwent data extraction. The PRISMA flow diagram for study inclusion is shown in Figure 1.

To extract data evidence, we created a table that shows the general characteristics of the selected studies, reporting information such as the authors, year of publication, country of origin, study aims, methods, participants and sample size, study design, type of analysis, and key findings relevant to the review questions.

Other recent reviews have also explored the role of artificial intelligence in obstetric care. For instance, a systematic review [42] of the application of machine-learning techniques to predicting delivery modes and obstetric complications highlighted the predominance of supervised learning methods and the lack of studies addressing postnatal care and reviewed the contributions of AI to pregnancy care, focusing on the prediction of conditions such as preeclampsia and gestational diabetes and emphasizing the potential of AI to enhance maternal health services [43]. Such reviews complement the present scoping review by providing a broader perspective on AI applications in obstetrics while reinforcing the relevance of addressing ethical considerations in the development and deployment of such technologies.

## 3. Results

Five articles entered the study, according to our aim. The main characteristics are summarized in Table 4. The selection described in the methods section revealed that all showed application in preterm birth, but also in hypertensive disorders of pregnancy and gestational diabetes. It is clear that preterm birth may be associated with restricted fetal growth, low birth weight, and small gestational age. However, none of these studies presented a model aimed particularly at placental abruption and stillbirth. One study also looked at maternal mortality. The articles studied are from continental countries, except for one, and none are European. All articles used the medical records of pregnant women followed in prenatal care or of children to predict preterm birth with some indicators and associated them with machine-learning (ML) algorithms.

Regarding Study 1, the predictive model based on the XGBoost shows potential for forecasting preterm birth, and particularly at the time of delivery, alanine aminotransferase (ALP), alpha-fetoprotein (AFP), albumin (ALB), hematocrit (HCT), total cholesterol (TC), alkaline phosphatase (ALT), platelets (PLT), height, diastolic blood pressure (DBP) and systolic blood pressure (SBP) are influential factors. It is a powerful approach that should be considered alongside other clinical and ultrasound data. Only a holistic approach increases the accuracy and reliability of the model. Data were derived from a single hospital, so it would be necessary to collect more medical information to expand the model to other populations, increasing its effectiveness. 

In Study 2, seven classic machine-learning models were used to classify the LBW and non-LBW records. Applying the weight rebalancing method, XGBoost had the highest recall score. Subsequent studies should be conducted across different regions and consider factors such as ethnicity, medical history, and socioeconomic status, but they can create healthcare policies and support clinicians with early prenatal interventions for pregnant women at the highest risk of LBW. Patients could be advised to have support for a healthy pregnancy with prenatal care, since this study also identified that those in less favorable conditions have a higher chance of having a baby with LBW.

Study 3 predicts maternal risk through adverse pregnancy events. Reducing maternal morbidity and mortality is part of the sustainable development goals. A comparison of ten ML algorithms demonstrated that the random forest (RF) method outperformed the other methods in predicting the risk level. RF averages various decision trees from multiple subsets and can overcome the issue of overfitting. The synthetic minority oversampling technique (SMOTE), applied to address an imbalanced dataset, was chosen over other methods. It applies principal component analysis (PCA) to solve the high multicollinearity. Without PCA, XGBoost was the second-best-performing algorithm. When using PCA, this position was occupied by K-nearest neighbors (KNN). RF is the top-performing algorithm for predicting maternal risk levels. No clinical features were included, so in the future, more clinical and lifestyle factors will be important to improve the model.

According to Study 4, the joint use of ML along with easily available variables from prenatal visits of New Mothers-to-Be (nuMoM2b) and ultrasound measurements, like cervical length and the pulsatility index, improves the model. At the first visit, 35 variables were recorded; at the second visit, 51 variables were available; and at the third visit, 73 variables were collected. Predictive assessments for preterm birth were found to be most effective during visit three (22–29 weeks of gestation), indicating that the timing and type of clinical data may hold more significance than sample size or computational complexity. While the model is based on traditional regression, which facilitates healthcare delivery, it is crucial to develop a more customized approach. With a sensitivity of 92.95% at visit three, it effectively identifies patients in need of intensive monitoring and early interventions. Moreover, using data from only the third visit, the model achieves predictive accuracy comparable to those utilizing data from all three visits. The model also shows high sensitivity in accurately detecting very and extremely preterm births during visit three.

Study 5’s analyses of six ML models were applied using large-scale survey data from preschool children, with key predictors identified through Shapley additive explanations (SHAP) on the XGBoost model. It demonstrated the impact of each feature on PTB prediction, with the best overall performance consistently and effectively across sets. The study also showed that the potential of ML in predicting PTB lies in its non-invasive nature and wide applicability. SHAP analysis revealed key predictors of PTB, such as multiple pregnancies, threatened abortion, and maternal age at conception.

To summarize, Table 5 shows the AI tool that each study used, the APO it focused on, its results, and the most important ethical considerations in each one. The integration of AI into maternal healthcare has emerged as a promising tool for predicting and managing APOs, such as preterm birth, hypertensive disorders, gestational diabetes, fetal growth restriction, and low birth weight. These conditions continue to represent significant global health challenges, often contributing to maternal and neonatal morbidity and mortality. AI offers the potential to enhance clinical decision-making by identifying high-risk pregnancies early, enabling timely interventions and better resource allocation.

### 3.1. AI Algorithms in APOs

Studies 1, 4, and 5 used AI mainly for preterm birth, while Study 2 used it primarily for low birth weight, and Study 3 for hypertensive disorders. Naturally, and because they were directly or indirectly related, other APOs were associated in each of the studies. The most used algorithm was XGBoost, but RF and KNN were also essential. Other resources, such as SMOTE and PCA, showed their applicability. Interestingly, SHAP was also used for explainability in Study 5. The truth is that they all improve APOs, regardless of which one or ones they work on. These algorithms were applied to improve risk prediction and support clinical decision-making, consistently improving the analyzed results.

Across recent studies, ML models such as XGBoost, RF, KNN, SMOTE, PCA, and interpretability tools like SHAP have been employed to analyze complex clinical, demographic, and biochemical data. These models have demonstrated substantial improvements in predictive accuracy and outcome classification when compared to traditional statistical approaches. XGBoost was consistently used across multiple studies due to its robustness in handling imbalanced datasets and its ability to capture non-linear relationships within large feature sets.

### 3.2. The Ethical Issues

Table 4 and Table 5 include not only the clinical outcomes but also the main ethical aspects discussed in each study, including concerns related to algorithmic bias, transparency, informed consent, and the impact on the doctor–patient relationship.

The integration of AI in this context is not without ethical challenges. The main concerns revolve around algorithmic bias, automated clinical decisions, model interpretation, data accuracy, informed consent, and transparency, especially in relation to underrepresented groups. Issues such as data privacy, regulation, and the impact of AI on medical trust are also highlighted.

Despite these obstacles, the five studies analyzed indicate that, with the proper handling of ethical issues, AI can be a valuable tool in improving perinatal care and promoting more informed and personalized decisions. Several studies have shown positive results from the application of AI algorithms in predicting APOs, such as preterm birth, hypertensive disorders, gestational diabetes, low birth weight, and fetal growth restriction. Models such as XGBoost, RF, KNN, and explanatory approaches such as SHAP have been used to improve diagnostic accuracy and support clinical decision-making. All the studies analyzed point to significant improvements in the results obtained with the use of these tools.

However, the incorporation of AI in maternal health raises complex ethical issues that go beyond generic concepts such as algorithmic bias or lack of transparency. A more situated analysis reveals critical challenges, such as obtaining informed consent in populations with low levels of literacy, something particularly relevant in contexts of social vulnerability. In these situations, clear communication about how the data will be used and how AI predictions will influence medical decisions becomes fundamental but is often neglected. Another central ethical dilemma is the conflict between AI-generated predictions and human clinical judgment. In high-risk scenarios, such as gestational complications, doctors may be faced with AI recommendations that challenge their experience or empirical assessment. This raises questions about professional responsibility, trust in the system, and the autonomy of clinical practice.

Additionally, there is an urgent need to consider the long-term implications for data privacy. Sensitive information related to pregnancy—including reproductive history, maternal and fetal health conditions, and genetic data—requires special protection. The possibility of future use of these data by insurance companies, government institutions, or other commercial entities raises legitimate concerns about ongoing consent and distributive justice. So, while AI algorithms have the potential to positively transform perinatal care, their implementation requires an ethical approach that is sensitive to the context of maternal health, incorporating robust safeguards and governance mechanisms that protect the rights, dignity, and trust of pregnant women.

## 4. Discussion

Artificial intelligence encompasses machine learning, including deep-learning techniques and, more recently, generative AI models [49,50]. The five studies analyzed explore the use of ML models in predicting obstetric outcomes, highlighting the potential of these tools to improve prenatal care and reduce maternal and child risks. Most of the studies are retrospective [44,45,47,48], leading to some kind of bias. Only one was quantitative [46]. Most of the studies do not have a topic in the article’s structure to discuss ethical issues, but such issues are mentioned in the discussion and limitations of the study. More emphasis should be placed on the ethical aspects; it is not enough to create functional models, but it is also important to understand the implications they have beyond the practical point of view. If they are to be applied professionally, it must be borne in mind that we are involving patients, their families, and health professionals.

### 4.1. AI Models in Obstetric Care

AI has shown considerable potential in predicting APOs such as preterm birth (PTB), low birth weight (LBW), gestational diabetes, and hypertensive disorders. Among the five studies analyzed, the most used models were XGBoost and RF, both showing strong predictive performance.

Study 1 highlighted the value of combining clinical and ultrasound data, with XGBoost accurately identifying key risk factors like blood pressure and biochemical markers. In Study 2, the application of data rebalancing techniques like the weighing method enhanced XGBoost’s performance in predicting LBW. Study 3 used RF, SMOTE, and PCA to mitigate class imbalance and multicollinearity, respectively, providing a robust framework for assessing maternal risk levels. Study 4 emphasized the importance of timing and quality in clinical data collection, demonstrating that early and accurate records are more impactful than sample size or computational complexity. Study 5 integrated SHAP into the XGBoost model, enhancing interpretability by explaining the contribution of each variable to the final prediction, which is crucial for clinical application.

Although the performance of these models is encouraging, it is important to note that most of the studies analyzed were retrospective and based on specific populations, which limits generalizability and calls for broader validation.

While this review primarily focuses on preterm birth, gestational hypertension, and gestational diabetes, it is important to acknowledge the limited analysis of other severe complications such as placental abruption and stillbirth. The scarcity of studies addressing these outcomes reflects a gap in current research. Notably, recent studies have begun to explore the application of machine-learning models in predicting stillbirth. A systematic review highlighted the potential of AI in stillbirth prediction, emphasizing the need for further development before clinical application. Incorporating such outcomes in future AI research is crucial, given their significant impact on maternal and fetal health [51].

### 4.2. Ethical Implications

In clinical contexts, the introduction of AI tools raises profound ethical dilemmas that extend beyond abstract concepts like bias or transparency. Consider a situation in which an AI model classifies a pregnant patient as high-risk for preterm birth based on aggregated population-level data. If the clinician’s own judgment, based on in-person evaluation and experience, contradicts this prediction, whose assessment should guide the decision? This scenario highlights the potential erosion of professional autonomy and accountability, especially if institutions begin to prioritize algorithmic recommendations over clinical expertise.

Furthermore, obtaining truly informed consent in settings with low health literacy presents a serious challenge. In many low-resource contexts, patients may not fully understand how their data are used, what AI predictions mean, or how these tools influence the care they receive. This creates a risk of “consent by default”, where the ethical principle of autonomy is undermined. To address this, AI implementation must be accompanied by clear, accessible explanations and culturally appropriate communication strategies. These concerns are particularly relevant in the field of maternal health, where decisions can have profound implications for both mother and child.

Despite the promising results, the ethical dimension of implementing AI in obstetrics remains underdeveloped in most studies, often relegated to brief mentions in the discussion or limitations. However, this aspect is critical, as these models directly impact patients, families, and healthcare providers.

Interpretability and transparency are also essential. Tools like SHAP offer pathways to understand AI decisions, but the clinical community must be trained to interpret these explanations correctly. Additionally, privacy and data governance must be prioritized, especially given the sensitive nature of reproductive health data. Concerns over long-term data use, potential misuse, and cybersecurity must be addressed through robust legal and regulatory frameworks.

The doctor–patient relationship is another ethical frontier. AI should support and not replace clinical judgment. Medical responsibility must remain with physicians, and any system that dehumanizes care or undermines clinical intuition risks compromising both trust and quality of care.

The ethical implications of integrating AI into maternal healthcare extend beyond concerns of algorithmic bias and transparency.

These ethical considerations may also be examined through the framework of classical bioethical principles. The principle of autonomy is challenged when informed consent is inadequately obtained or when patients, particularly those with limited health literacy, are not fully aware of how artificial intelligence influences clinical decisions. The principle of beneficence supports the integration of AI in obstetric care insofar as it contributes to the early identification of risk and the improvement of maternal and neonatal outcomes. Conversely, the principle of non-maleficence may be compromised if AI systems introduce diagnostic errors, reinforce clinical overdependence, or operate without sufficient interpretability. Finally, the principle of justice is central to addressing disparities in data representation and access to AI-driven interventions. The risk of exacerbating existing inequities must be actively mitigated through inclusive model development and equitable implementation strategies [52].

### 4.3. Challenges and Opportunities

One of the central challenges in implementing AI in maternal healthcare is the limited generalizability of current models. Many studies are based on data from single institutions or high-income regions, failing to represent the diversity of global populations. This limitation compromises the applicability of predictive algorithms in underserved settings, where healthcare systems often face structural limitations and digital inequalities. Additionally, the omission of key clinical and lifestyle variables, such as those observed in Study 3, reduces the capacity of these models to grasp multifactorial risks fully. Language, health literacy, and socioeconomic inequality affect data collection and limit the ethical and practical viability of AI tools in real-world clinical contexts.

Despite these challenges, AI presents significant opportunities. Properly developed and implemented, AI enables earlier and more personalized risk assessments, facilitating timely clinical interventions and improving resource allocation. Precision medicine becomes more attainable as algorithms can flag high-risk pregnancies with increasing accuracy. Another major opportunity lies in promoting interoperability through the creation of standardized, shareable datasets. This can reduce the administrative burden on health professionals, strengthen maternal care systems, and foster more inclusive and collaborative research across borders.

However, the rapid evolution of AI continuously forces us to rethink strategies and regulatory approaches. As AI systems grow more complex, their integration into healthcare must be guided by strong ethical principles and a commitment to human dignity and autonomy. Research integrity, encompassing transparency, impartiality, and accountability throughout the entire development and application process, is essential, particularly in areas with profound social impact [53].

According to the EU Artificial Intelligence Act (EU AI Act), most AI systems used in obstetrics would fall into the high-risk category, given their potential to influence clinical decisions and affect patient safety [54]. These systems are therefore subject to strict requirements, including continuous monitoring, documentation, and explainability. While some may fall into the limited-risk category, if they only assist decision-making without directly influencing it, they still require clear user communication about functionality and limitations.

Lastly, informed consent must be more than a formality: it should act as a key transparency mechanism that ensures patients are not only aware of AI’s involvement but also understand it. Especially in maternal healthcare, preserving humanized medicine and the doctor–patient relationship is not optional—it is essential [55,56].

### 4.4. Comparison with Other Surveys and Studies

Compared to broader reviews and international surveys, this analysis shares common findings regarding the efficacy of tree-based models like XGBoost and RF. However, while many studies confirm the performance of these models, few delve deeply into the ethical and systemic implications of their clinical integration.

Other surveys, particularly those aligned with the WHO (World Health Organization) or large consortia such as the National Institutes of Health (NIH), place greater emphasis on the socioeconomic determinants of health and the global digital divide, which are less prominent in the five studies analyzed here. Additionally, these larger reviews often advocate for cross-national collaboration and policy alignment, recognizing that regulation must evolve in step with technological advancements.

The present analysis underscores a critical need for more interdisciplinary studies that evaluate model performance and incorporate robust ethical frameworks, legal perspectives, and social sciences to guide implementation.

The provision for liability for damage attributable to the application of AI must be considered. AI must be scalable, growing with its use, and should work correctly over time, and it is difficult for regulation to follow this development.

This study aimed to evaluate AI’s impact on adverse pregnancy outcomes APOs. The results confirm that AI tools support clinical decision-making and individualize patient care through earlier and more precise risk assessments. The research question concerning the ethical challenges associated with AI in obstetrics is also addressed, with findings underscoring the need for transparency, regulation, and the preservation of the doctor–patient relationship.

Although advances in the application of AI in predicting APOs are promising, it is important to recognize some limitations that compromise the generalizability of the results and their applicability in global contexts. Firstly, the lack of studies from regions such as Europe, Africa, and South Asia highlights the uneven distribution of data. This limitation restricts the external validity of the models and raises concerns about the representativeness of populations with significantly different socioeconomic, cultural, and health infrastructure profiles. In low-income areas, where maternal mortality rates are high, the challenges related to data collection and quality can be even more pronounced, compromising the effectiveness of algorithms trained in more favored contexts.

Additionally, although the study aimed to investigate a variety of APOs, the actual analysis focused mainly on preterm birth, gestational hypertension, and gestational diabetes. This methodological choice may have been influenced by the availability and consistency of clinical data relating to these outcomes, but it ultimately neglects serious complications such as placental abruption and stillbirths. These conditions, which often present a high risk of fatal outcomes, demand special attention as they may require immediate and strategic interventions.

Therefore, future research must broaden the scope of the analysis, incorporating these critical outcomes and considering the particularities of different regional and socioeconomic contexts. Expanding the range of indicators to be analyzed could not only improve the accuracy of predictive models but also contribute to designing health policies that are more inclusive and adapted to local realities. In addition, the integration of data from different regions can facilitate the development of more robust algorithms, capable of dealing with the variability inherent in health systems around the world.

Finally, improving data collection and analysis methods is key to ensuring that AI models meet the specific needs of vulnerable populations. This integrated and diverse approach will allow for a better understanding of risk factors. It will contribute to the formulation of more effective intervention strategies, promoting equity in the provision of maternal and newborn health care.

## 5. Conclusions

The integration of artificial intelligence into maternal healthcare presents a significant opportunity to improve the prediction and management of adverse pregnancy outcomes (APOs), particularly preterm birth, hypertensive disorders, gestational diabetes, fetal growth restriction, and low birth weight. The five studies analyzed in this scoping review demonstrate that machine-learning models, particularly XGBoost and random forest, can support earlier diagnosis, enhance clinical decision-making, and personalize prenatal care.

However, the analysis also reveals important limitations. Despite the intention to cover a broad spectrum of APOs, key complications such as placental abruption and stillbirth remain unaddressed in the included literature.

Additionally, the generalizability of findings is constrained by the geographic concentration of studies and the underrepresentation of vulnerable populations.

Ethical concerns such as algorithmic bias, informed consent, and data privacy demand urgent attention, particularly in low-resource settings where disparities in health literacy and digital access persist. To ensure AI contributes positively to maternal health, its deployment must be guided by robust ethical principles, interdisciplinary collaboration, and strict regulatory oversight. AI must be transparent, explainable, and continuously validated in diverse populations. It should function as a tool that supports, rather than replaces, clinical expertise and the doctor–patient relationship.

Looking ahead, the challenge is not only technological but profoundly human. The future of maternal care must balance innovation with equity, automation with empathy, and data with dignity. Artificial intelligence has the potential to humanize healthcare, but only if implemented with responsibility, reflection, and respect for the people it aims to serve.

## Figures and Tables

**Table 1:** Query box for SCOPUS search.

SCOPUS
((TITLE-ABS-KEY (pregnant) OR TITLE-ABS-KEY (pregnancy))) AND ((TITLE-ABS-KEY (pregnancy AND risk) OR TITLE-ABS-KEY (adverse AND pregnancy AND outcomes) OR TITLE-ABS-KEY (hypertensive AND disorders AND of AND pregnancy) OR TITLE-ABS-KEY (gestational AND diabetes AND mellitus) OR TITLE-ABS-KEY (preterm AND birth) OR TITLE-ABS-KEY (fetal AND growth AND restriction) OR TITLE-ABS-KEY (low AND birthweight) OR TITLE-ABS-KEY (small AND for AND gestational AND age AND newborn) OR TITLE-ABS-KEY (placental AND abruption) OR TITLE-ABS-KEY (stillbirth))) AND ((TITLE-ABS-KEY (artificial AND neural AND networks) OR TITLE-ABS-KEY (artificial AND intelligence) OR TITLE-ABS-KEY (machine AND learning))) AND ((TITLE-ABS-KEY (ethics) OR TITLE-ABS-KEY (ethical AND issues) OR TITLE-ABS-KEY (bioethics))) AND PUBYEAR > 2019 AND PUBYEAR < 2025.

**Table 2:** Query box for PubMed search.

PubMed
**query #1** (pregnant) OR (pregnancy)
**query #2** pregnancy risk) OR (adverse pregnancy outcomes) OR (hypertensive disorders of pregnancy) OR (gestational diabetes mellitus) OR (preterm birth) OR (fetal growth restriction) OR (low birthweight) OR (small for gestational age newborn) OR (placental abruption) OR (stillbirth)
**query #3** (ethics) OR (ethical issues) OR (bioethics)
**query #4** (artificial neural networks) OR (artificial intelligence) OR (machine learning)
**query #5** query #1 AND query #2 AND query #3 AND query #4

**Table 3:** Query box for Web of Science search.

Web of Science
query #1 (pregnant (All Fields) or pregnancy (All Fields)
query #2 pregnancy risk (All Fields) or adverse pregnancy outcomes (All Fields) or hypertensive disorders of pregnancy (All Fields) or gestational diabetes mellitus (All Fields) or preterm birth (All Fields) or fetal growth restriction (All Fields) or low birthweight (All Fields) or small for gestational age newborn (All Fields) or placental abruption (All Fields) or stillbirth (All Fields)
query #3 ethics (All Fields) or ethical issues (All Fields) or bioethics (All Fields)
query #4 artificial neural networks (All Fields) or artificial intelligence (All Fields) or machine learning (All Fields)
query #5 #4 AND #3 AND #2 AND #1

**Table 4:** Data extraction and synthesis.

Study, First Author, Country, Year Published	Title	Setting of Data Source	Purpose	Design of the Study	Data Collection Tools	Participants	Outcomes	Results	Ethical Discussion	Key Findings
**Study 1** Yu Chen China, 2024 [44]	Development and validation of a spontaneous preterm birth risk prediction algorithm based on maternal bioinformatics: A single-center retrospective study.	Pregnant women who registered for prenatal care and gave birth at the outpatient department of Hangzhou Women’s Hospital.	* The primary objective of this study is to identify all potential risk factors for preterm birth using clinical laboratory big data and to select the most significant factors from this dataset to establish an accurate risk prediction range. * The secondary aim of this study is to validate and assess the predictive accuracy of the model containing the ten most relevant features identified in the previous step. The goal is for this tool to reliably evaluate and quantify individual preterm birth risks, offering guidance for optimal clinical management in each case and providing a foundation for the development of future clinical assessment tools.	Observational, retrospective study.	Examined the clinical data of pregnant women who registered for prenatal care and gave birth at Hangzhou Women’s Hospital’s outpatient department from January 2019 to December 2022.	n = 3.082 Pregnant women were classified into two groups: the sPTB (spontaneous preterm birth) group consisting of those who delivered before 37 weeks of gestation, and the full-term group, comprising women who delivered at or after 37 weeks.	* Preterm birth. * Hypertensive disorders of pregnancy (gestational hypertension, preeclampsia). * Gestational diabetes.	A total of 24 indicators showing significant differences. Regarding preterm birth risk prediction, the extreme gradient boosting (XGBoost) algorithm exhibited the best performance, achieving an AUC ROC of 0.89 (95% CI: 0.88–0.90). The ten most important indicators were alanine aminotransferase (ALP), alpha-fetoprotein (AFP), albumin (ALB), hematocrit (HCT), total cholesterol (TC), diastolic blood pressure (DBP), alkaline phosphatase (ALT), platelets (PLT), height, and systolic blood pressure (SBP), which are important to an accurate predictive model. The model demonstrated consistent performance across both the training and validation datasets, achieving AUC values of 0.93 and 0.87. The external testing set also displayed strong performance with an AUC of 0.79.	* The importance of AI predictions with clinical decision and as a supportive tool. * Bias and interpretability.	* The study findings suggest that at the time of delivery, ALP, AFP, ALB, HCT, TC, DBP, ALT, PLT height, and SBP are significant factors influencing sPTB. * The predictive model utilizing the XGBoost algorithm shows promise for forecasting preterm birth in early pregnancy and could serve as a reference for the clinical implementation of personalized risk prediction for sPTB.
**Study 2** Yang Ren USA, 2023 [45]	Issue of Data Imbalance on Low-Birth-Weight Baby Outcomes Prediction and Associated Risk Factors Identification: Establishment of Benchmarking Key Machine-learning Models with Data Rebalancing Strategies.	Birth records from the first quarter of 2015 to the first quarter of 2021 from a large health care system in a southeast state of the United States.	* Establish several key benchmarking machine-learning (ML) models to predict low birth weight (LBW) and systematically apply different rebalancing optimization methods to a large-scale and extremely imbalanced all-payer hospital record data set that connects mother and baby data at a state level in the United States. * Analysis to identify the most contributing features in the LBW classification task, which can aid in targeted intervention.	Retrospective cohort study.	Large-scale, dataset that links mother and baby births.	n = 266.687 (birth records).	* Low birth weight. * Preterm birth. * Hypertensive disorders of pregnancy (gestational hypertension, preeclampsia). * Gestational diabetes.	Various data rebalancing methods improved the prediction performance of the LBW group substantially. From the feature importance analysis, maternal race, age, payment source, sum of predelivery emergency department and inpatient hospitalizations, predelivery disease profile, and different social vulnerability index components were important risk factors associated with LBW.	* Bias. * Inaccuracy. * Clinical decision-making. * AI as support tool. * Explainability and transparency.	Useful ML benchmarks to improve birth outcomes in the maternal health domain. They are informative to identify the minority class (e.g., LBW) based on an extremely imbalanced data set, which may guide the development of personalized LBW early prevention, clinical interventions, and statewide maternal and infant health policy changes.
**Study 3** Sulaiman Salim Al Mashrafi Omã, 2024 [46]	Predicting Maternal Risk Level using Machine-Learning Models.	* Oman’s Civil Registration and Vital Statistics system. * Data from different available sources in Oman.	To investigate the potential of machine-learning algorithms in predicting maternal risk levels.	Quantitative research design.	Data is routinely gathered by Oman’s health information system, where each birth or death is reported directly from the health institution.	n = 402 (reported maternal deaths in Oman from 1991 to 2023).	* Hypertensive disorders of pregnancy. * Gestational diabetes. * Preterm birth.	The findings showed that the random forest (RF) model surpassed the other methods in predicting the risk levels (low or high), achieving an accuracy of 75.2%, a precision of 85.7%, and an F1-score of 73% after applying principal component analysis (PCA).	* Bias - AI can reinforce disparities. * Certain groups are misdiagnosed. * Need for representative data collection. * Informed consent and data protection. * Lack of patient and doctor confidence. * Regulatory oversight.	* RF outperforms other algorithms in predicting the risk level, with K-nearest neighbors (KNN) following closely behind. * Future work should incorporate additional clinical and lifestyle factors pertaining to the mother, as well as factors related to the fetus and the baby, to enhance and refine the model for predicting maternal risk levels.
**Study 4** Chenyan Huang USA, 2024 [47]	Predicting Preterm Birth using Electronic Medical Records from Multiple Prenatal Visits.	Nulliparous women carrying singleton pregnancies, recruited from eight clinical centers associated with research institutions. * Case Western Reserve University. * Columbia University. * Indiana University. * Magee-Women’s Hospital. * Northwestern University. * University of California-Irvine. * University of Pennsylvania. * University of Utah.	* Determine maternal characteristics, including genetics, epigenetics, and physiological response to pregnancy and environmental factors that influence or predict adverse pregnancy outcomes (APOs). * Identify specific aspects of placental development and function that lead to APO. * Characterize genetic, growth, and developmental parameters of the fetus that are associated with APO.	Retrospective cohort study.	Data were collected through interviews, self-administered questionnaires, clinical assessments, ultrasounds, and a review of medical records during four planned study visits.	n = 10.038 (nulliparous women).	* Preterm birth. * Low birth weight. * Hypertensive disorders of pregnancy. * Gestational diabetes.	Machine-learning models improved the prediction accuracy of PTB, especially in very preterm (<32 weeks) and extreme-preterm (<28 weeks) cases. From visit three, we can start predicting preterm birth. The model also exhibits high sensitivity in accurately identifying very and extreme-preterm births during the third visit, indicating that positive cases in these two subgroups can be correctly predicted, enabling timely and targeted clinical interventions.	* Bias. * Underrepresented groups. * Transparency. * Explainability and responsible use.	* This study shows that predictive evaluations for preterm birth are most accurate during visit three (22–29 gestational weeks), with the AUC increasing from 0.6161 at visit one to 0.7087 at visit three. * The results emphasize the significance of ultrasound measurements and suggest that integrating machine-learning-based risk assessments and routine ultrasounds in late pregnancy into prenatal care could enhance maternal and neonatal outcomes by facilitating timely interventions for high-risk women.
**Study 5** Liwen Ding China, 2024 [48]	Prediction of Preterm Birth using Machine Learning: a Comprehensive Analysis Based on Large-Scale Preschool Children Survey Data in Shenzhen of China.	235 kindergartens in Longhua District, Shenzhen, China.	To develop and evaluate six machine-learning models to predict PTB using large-scale children survey data from Shenzhen, China, and to identify key predictors through Shapley additive explanations (SHAP) analysis.	Observational retrospective study.	The data were collected through a self-administered online structured questionnaire, completed under the supervision of childcare practitioners and teachers.	n = 84.050 mother–child pairs (collected in 2021 and 2022).	* Preterm birth. * Hypertensive disorders of pregnancy. * Gestational diabetes. * Fetal growth restriction.	The XGBoost model exhibited the highest overall performance, achieving area under the receiver operating characteristic curve (AUC) scores of 0.752 and 0.757 in the validation and test sets, respectively, along with favorable calibration and clinical applicability. Key predictors identified included multiple pregnancies, threatened abortion, and maternal age at conception. SHAP analysis emphasized the positive impacts of multiple pregnancies and threatened abortion, while highlighting the negative effects of micronutrient supplementation on PTB.	* Bias, especially in marginalized populations * The “black box” and how risk predictions are generated. * Lack of regulation and the slow adoption of frameworks. * Data privacy and decision support.	* ML models, particularly XGBoost, demonstrate strong potential in accurately predicting PTB and identifying critical risk factors. * These findings highlight the potential of ML to improve clinical interventions, personalize prenatal care, and guide public health initiatives.

**Table 5:** Outcomes assessed in each study.

Included	APO	AI	Ethical Issues	Result
**Study 1**	Preterm birth Hypertensive disorders Gestational diabetes	XGBoost	Bias Clinical decision Interpretability	Improvement
**Study 2**	Low birth weight Preterm birth Hypertensive disorders Gestational diabetes	XGBoost	Bias Inaccuracy	Improvement
**Study 3**	Hypertensive disorders Gestational diabetes Preterm birth	RF SMOTE PCA	Bias Informed consent Doctor confidence	Improvement
**Study 4**	Preterm birth Low birth weight Hypertensive disorders Gestational diabetes	KNN	Underrepresented groups Transparency	Improvement
**Study 5**	Preterm birth Hypertensive disorders Gestational diabetes Fetal growth restriction	XGBoost SHAP	Bias Regulation Data privacy Decision support	Improvement

## Data Availability

Not applicable.

## Supplementary Materials

The following supporting information can be downloaded at https://www.mdpi.com/article/10.3390/jcm14113860/s1, PRISMA-ScR Checklist. Reference [38] is cited in the supplementary materials.

## Abbreviations

The following abbreviations are used in this manuscript:

AI	Artificial intelligence
AFP	Alpha-fetoprotein
ALB	Albumin
ALP	Alanine phosphatase
ALT	Alkaline aminotransferase
APOs	Adverse pregnancy outcomes
DBP	Diastolic blood pressure
ELBW	Extremely low birth weight
EU AI Act	European Regulation on Artificial Intelligence
FGR	Fetal growth restriction
GDM	Gestational diabetes mellitus
HDP	Hypertensive disorders of pregnancy
HCT	Hematocrit
KNN	K-nearest neighbors
LBW	Low birth weight
ML	Machine learning
NIH	National Institutes of Health
nuMoM2b	New Mothers-to-Be
OGTT	Oral glucose tolerance test
PCA	Principal component analysis
PLT	Platelets
PRISMA-SCR	PRISMA-ScR Preferred Reporting Items for Systematic Reviews and Meta-analyses Extension for Scoping Reviews
PTB	Preterm birth
RF	Random forest
SDGs	Sustainable development goals
SGA	Small for gestational age
SMOTE	Synthetic minority oversampling technique
SBP	Systolic blood pressure
TC	Total cholesterol
VLBW	Very low birth weight
WHO	World Health Organization
